# The impact of visuospatial and executive function on activity performance and outcome after robotic or conventional gait training, long-term after stroke—as part of a randomized controlled trial

**DOI:** 10.1371/journal.pone.0281212

**Published:** 2023-03-09

**Authors:** Maria Bergqvist, Marika C Möller, Martin Björklund, Jörgen Borg, Susanne Palmcrantz

**Affiliations:** 1 Department of Rehabilitation Medicine, Danderyd University Hospital, Stockholm, Sweden; 2 Department of Clinical Sciences, Danderyd Hospital, Karolinska Institutet, Stockholm, Sweden; 3 Department of Community Medicine and Rehabilitation Physiotherapy, Umeå University, Umeå, Sweden; BG-Universitatsklinikum Bergmannsheil, Ruhr-Universitat Bochum, GERMANY

## Abstract

**Introduction:**

Visuospatial and executive impairments have been associated with poor activity performance sub-acute after stroke. Potential associations long-term and in relation to outcome of rehabilitation interventions need further exploration.

**Aims:**

To explore associations between visuospatial and executive function and 1) activity performance (mobility, self-care and domestic life) and 2) outcome after 6 weeks of conventional gait training and/or robotic gait training, long term (1–10 years) after stroke.

**Methods:**

Participants (n = 45), living with stroke affecting walking ability and who could perform the items assessing visuospatial/executive function included in the Montreal Cognitive Assessment (MoCA Vis/Ex) were included as part of a randomized controlled trial. Executive function was evaluated using ratings by significant others according to the Dysexecutive Questionnaire (DEX); activity performance using 6-minute walk test (6MWT), 10-meter walk test (10MWT), Berg balance scale, Functional Ambulation Categories, Barthel Index and Stroke Impact Scale.

**Results:**

MoCA Vis/Ex was significantly associated with baseline activity performance, long-term after stroke (*r* = .34-.69, *p* < .05). In the conventional gait training group, MoCA Vis/Ex explained 34% of the variance in 6MWT after the six-week intervention (*p* = 0.017) and 31% (*p* = 0.032) at the 6 month follow up, which indicate that a higher MoCA Vis/Ex score enhanced the improvement. The robotic gait training group presented no significant associations between MoCA Vis/Ex and 6MWT indicating that visuospatial/executive function did not affect outcome. Rated executive function (DEX) presented no significant associations to activity performance or outcome after gait training.

**Conclusion:**

Visuospatial/executive function may significantly affect activity performance and the outcome of rehabilitation interventions for impaired mobility long-term after stroke and should be considered in the planning of such interventions. Patients with severely impaired visuospatial/executive function may benefit from robotic gait training since improvement was seen irrespective of visuospatial/executive function. These results may guide future larger studies on interventions targeting long-term walking ability and activity performance.

**Trial registration:**

clinicaltrials.gov (NCT02545088) August 24, 2015.

## Introduction

Stroke is one of the five most common causes of death and functional impairment in the world [1]. The consequences, including impaired motor and sensory function, pain, abnormal muscle tone and cognitive and communicative impairments, vary depending on the location and severity of the stroke [2]. The recovery after stroke is most pronounced during the first weeks to months after stroke onset, but further improvements may be seen long-term (>6 months) after stroke [3–5].

Cognitive impairments are commonly reported after stroke [6]. In a study including participants >50 years of age, 83% had cognitive impairments three months after stroke, most commonly related to executive function, memory and visuospatial function [7]. Executive functions are considered complex and include high level monitoring of cognitive, emotional and behavioral functions, involved in planning, executing and self-evaluation during goal-directed and future-oriented behavior [8]. This function includes aspects of self-regulation, initiation of activities, utilization of feedback, problem-solving and cognitive flexibility [8]. Visuospatial function, on the other hand, involves the ability to identify, perceive and interpret visual information and spatial relations, including the ability to navigate in space and interpret visual distances, movements, relations as well as the perception of time [9].

A number of studies have reported associations between cognition and activity performance after stroke, as well as cognition in the acute stage being an important predictor of both cognitive impairments and activity performance long term [10–14]. Cognitive impairments combined with poor motor recovery have been shown to increase the risk of poor health-related quality of life [10]. Furthermore, associations between executive function and balance, mobility, dependence in activities of daily living (ADL) and rehabilitation participation in the acute or subacute stage after stroke have been presented [13, 15–18]. Visuospatial function has shown to be a predictor of community mobility and instrumental ADL long term after stroke [14, 19, 20]. In above mentioned studies, insufficient measures of cognition in participants with mild stroke, along with a lack of longitudinal assessments hampers the generalizability of the results. This highlights the need for studies evaluating the associations between standardized assessments of cognitive function and activity performance, including specific exercise regimes. It is plausible that executive and visuospatial functions may be important in activity performance and particularly gait, because of its involvement in initiation of activities, problem-solving and interpreting visual distances, movements and relations of the body and environment. However, most studies are executed in the acute or subacute stages, leaving a gap in the long-term phase after stroke [14–17, 20].

To significantly improve gait function, short- and long-term after stroke, evidence indicate that effective gait training should be task-oriented and intensive [21]. Robotic gait training has been developed to enhance the provision of task-specific training, by enabling a maximization of repetitive gait movements and assistance when needed [22–24]. Several studies have evaluated the outcome of robotic gait training, showing effects on walking capacity,—velocity and -independence as well as gait pattern function, comparable to control groups receiving conventional gait training [23–28]. However, one aspect that may affect the generalizability of these study results was the exclusion of participants with moderate/severe cognitive impairments [23, 25].

Moreover, to our knowledge, no studies have evaluated the impact of visuospatial and executive function on the effect of specific gait interventions. Thus, the aims of this study were achieved by exploring the potential associations between visuospatial and executive function, and 1) aspects of activity performance (mobility, self-care and domestic life), as well as 2) effects on mobility outcome after robotic and/or conventional gait training, in persons living with hemiplegia affecting walking ability long-term after stroke, followed up after the intervention (6 weeks) and at 6 months.

## Method

### Design and implementation

This study is based on analyses as part of a randomized controlled trial (RCT) [29], conducted at the University Department of Rehabilitation Medicine, Danderyd Hospital and Department of Clinical Sciences at Karolinska Institutet in Stockholm, Sweden. The study was approved by the Swedish Ethical Review Authority (2015/1216-31) as a multicenter trial with the ordinal scale, Functional Ambulation Category, as primary outcome and 54 participants/site to reach statistical power. Before study start, the planned 3 study sites were reduced to 1, due to limited access to robotic suits for gait training. The 6-minute walk test (6MWT) was changed from secondary to primary outcome, to allow more sensitive analyses of changes in walking (48 participants, to reach statistical power, please see the statistics section below). The study was registered with these changes at ClinicalTrials.gov: NCT02545088 prior to study start.

The RCT was single blinded, with an assessor blinded to group allocation. Randomization was performed according to block randomization (prepared by a statistician not otherwise involved in the study, using the SAS system, including the variables: blocks of three, treatment and patient). A nurse not otherwise involved in the study randomized the participants after the baseline testing, by pulling the participant’s ID-number one by one from a prepared envelope and placing them in the order of the block starting at the top of each block. The result of the randomization was photographed and saved in the study file. Participants were randomized into three groups 1) One intervention group received gait training with the exoskeleton Hybrid Assistive Limb (HAL) as well as conventional gait and mobility training (HAL-group), 2) A second intervention group received conventional gait and mobility training (Conventional group), and 3) a control group, continued with their usual activities (Control group). The two intervention groups were dose-matched to out rule any variations in intensity and scheduled three times a week for six weeks, (18 sessions). In the HAL-group each session was scheduled for a maximum of 60 minutes of gait training with HAL, plus a maximum of 30 minutes of conventional gait and mobility training to enhance generalizability of the acquired skills to everyday life activities. The HAL was used on a treadmill with a safety harness including body weight support (pre-set to 9 kg) to unburden the weight of the HAL-suit. HAL can be individually set to support movements of the hip and knee joints and includes a hybrid system with a voluntary mode where the level of assistance is individually adjusted and triggered by muscle contractions detected by surface- electromyography (EMG) [30]. To optimize training intensity, the level of assistance from the HAL and the walking speed on the treadmill was continuously adjusted, as tolerated by the participant and as the participant improved. In the conventional group, each session was scheduled for a maximum of 90 minutes of gait and mobility training with a physiotherapist. The conventional training included individualized, challenging exercises targeting an improved walking ability, such as overground walking on varying surfaces with and without walking aid, treadmill walking, weightbearing on the paretic leg, motor training of the lower extremity, and mobility tasks.

### Participants

Eligible participants for the RCT were aged 18–70 years, had suffered a first ever ischemic or hemorrhagic stroke 1–10 years earlier and were living with stroke related hemiparesis in the lower extremity, affecting walking ability. Participants were recruited from rehabilitation units, in collaboration with physiotherapists in outpatient care in the Stockholm region. Written and oral information was provided to the eligible participants before a written consent was given. Inclusion and exclusion criteria are further described in the published RCT [29]. In the current analysis of data collected in the RCT, one exclusion criteria was added. Participants unable to perform the Trail Making B task, related to impaired verbal understanding and/or difficulties in interpreting letters and numbers due to severe aphasia, were excluded. The Trail Making B task is included in the Montreal Cognitive Assessment (MoCA) visuospatial/executive domain (MoCA Vis/Ex), further described in the Assessment methods section below.

### Data collection

Data for the RCT was collected between October 2015 and March 2020 [29]. The assessments were conducted by a blinded assessor, at baseline (M1), after six weeks (M2) as well as six (M3) and twelve (M4) months. In this analysis, M4 data were not included, due to the number of dropouts at M4 in the study sample.

### Assessment methods

#### Measures of visuospatial and executive function

For clinical assessment of visuospatial and executive function, the MoCA Vis/Ex was used [31]. The MoCA Vis/Ex consists of three tasks: the Trail Making B task, the three-dimensional cube copying task and the clock-drawing task [maximum score 5, a lower score indicating a higher degree of impairment] [31].

To assess rated executive function, the Dysexecutive Questionnaire (DEX), included in the Behavioral Assessment of Dysexecutive Syndrome (BADS) test battery, was used [32]. The DEX contains 20 items evaluating daily executive problems of patients with brain damage in their everyday routine [scored on a 5-point scale from “Never” to “Very often”, total score 0–80 points, a higher score indicating a higher level of executive dysfunction] [32]. In the present study, the scoring was made by a family member or close friend, since the assessor had no previous knowledge of the participants. Thus, the DEX ratings made by the significant other are henceforth defined DEX-SO.

#### Measures of mobility

To assess mobility, the following clinical tests were used: 6MWT, the primary outcome, to evaluate walking distance during a 6 minute walk [Minimally Clinical Important Difference (MCID) in a stroke population: 34.4 meters] [33, 34]; 10-metre walk test (10MWT) to measure walking speed [result in meters/second, MCID 0.06 m/s] [35, 36] and Berg balance scale (BBS) to evaluate static and dynamic balance [scoring 0–56 with a higher score indicating a better performance, MCID 2.7 points] [37]. In addition, the Functional Ambulation Category (FAC) was used to evaluate independence in ambulation [ranging from 0 (Non-functional ambulator) to 5 (Independent ambulator)] and the mobility subscale number 6, included in the Stroke Impact Scale (SIS), hereby named SIS-Mob to evaluate self-perceived mobility [total score 0 (maximal limitation)– 100 (no limitation)] [38, 39].

#### Measures of self-care and domestic life

Self-perceived performance of activities in daily life was evaluated using subscale 5, included in the SIS, hereby called SIS-ADL [total score ranging from 0 (maximal limitation)– 100 (no limitation)] [39]. Additionally, self-perceived independence in mobility and personal care was evaluated with the Barthel Index (BI) [total score ranging from 0 (fully dependent) to 100 (independent) [40].

### Statistics

The power of the RCT was calculated using F-test for One-Way ANOVA, with the 6MWT as a primary outcome, based on results from a previous study on perceived and measured change in walking distance in the long-term phase after stroke [34]. With a significance level of 0.05 and a power of 80%, a total of 48 participants were included in the RCT. Previous studies, including 28–63 participants, have reported low, moderate and strong correlations (*r* = .25 – .60) between cognition and measures of activity performance, with the significance level of 0.05 [13, 41, 42]. Hence, it would be considered likely to detect at least low to moderate correlations with approximately 30–45 participants, as expected in the current study.

Analyses of the collected data were carried out using the SPSS Analytics version 22 [43]. Descriptive statistics were presented as frequency and percentage for nominal data and median and interquartile range (IQR) for ordinal and not normally distributed data (detected with the Shapiro-Wilks test). Baseline (M1) data from all three groups, were used to explore the associations between visuospatial and executive function and aspects of mobility, self-care and domestic life. To explore associations between visuospatial and executive function and differences in mobility outcome (6MWT, 10MWT, FAC, BBS), or accomplished walking distance (6MWT) according to group allocation, data from the two intervention groups at M1 and M2 were used to evaluate effects at 6 weeks (ΔM2-M1), and M1 and M3 to evaluate effects at 6 months (ΔM3-M1). Associations were visualized with scatterplots, including MCID for interpretation of the result and the clinical importance [44]. Correlation coefficients were calculated using Spearman’s rho, since data was not normally distributed. To interpret the strength of the correlation coefficients, the cut-offs by Cohen were used: *r* = 0.10–0.29 low, *r* = 0.30–0.49 moderate, *r* = 0.50–1.0 strong [45]. To control for expected false discoveries due to multiple comparisons (Type-1 error), an adjusted alpha-level was calculated according to False Discovery Rate (FDR) by Benjamini and Hochberg [46].

To further interpret the statistically significant associations, linear regression analyses were carried out to calculate the coefficient of determination (*r*^2^), with the visuospatial and executive measures as independent variables, and the outcomes of mobility, self-care and domestic life, as dependent variables. All dependent variables except FAC were included in the linear regression analyses, as they were considered continuous, and fulfilled the requirements of linear regression analysis, including normality checked using Normal P-P Plot of Regression Standardized Residual [47, 48]. In the analysis including FAC, an ordinal regression (logit) analysis was performed. Additionally, to control for potential covariates, multivariable linear regression analyses were carried out, including baseline characteristic with *p*≤0.1, identified in univariate regression analyses. In the analysis of the association between visuospatial and executive function and activity performance, four independent variables fulfilled the criteria for inclusion (MoCA Vis/Ex, age, gender, stroke severity according to National Institutes of Health Stroke Scale (NIHSS), as well as three in the analysis of association between visuospatial and executive function and effect on mobility outcome (MoCA Vis/Ex, gender, NIHSS), and one (MoCA Vis/Ex) for the association between visuospatial and executive function and accomplished walking distance according to group allocation [49]. In the latter analysis the MoCA Vis/Ex was dichotomized based on an analysis of the scatterplots, with a cut-off set at 3 points (MoCA Vis/Ex 0–2 = Lower score, MoCA Vis/Ex 3–5 = Higher score).

## Results

### Participants

**Fig 1:**
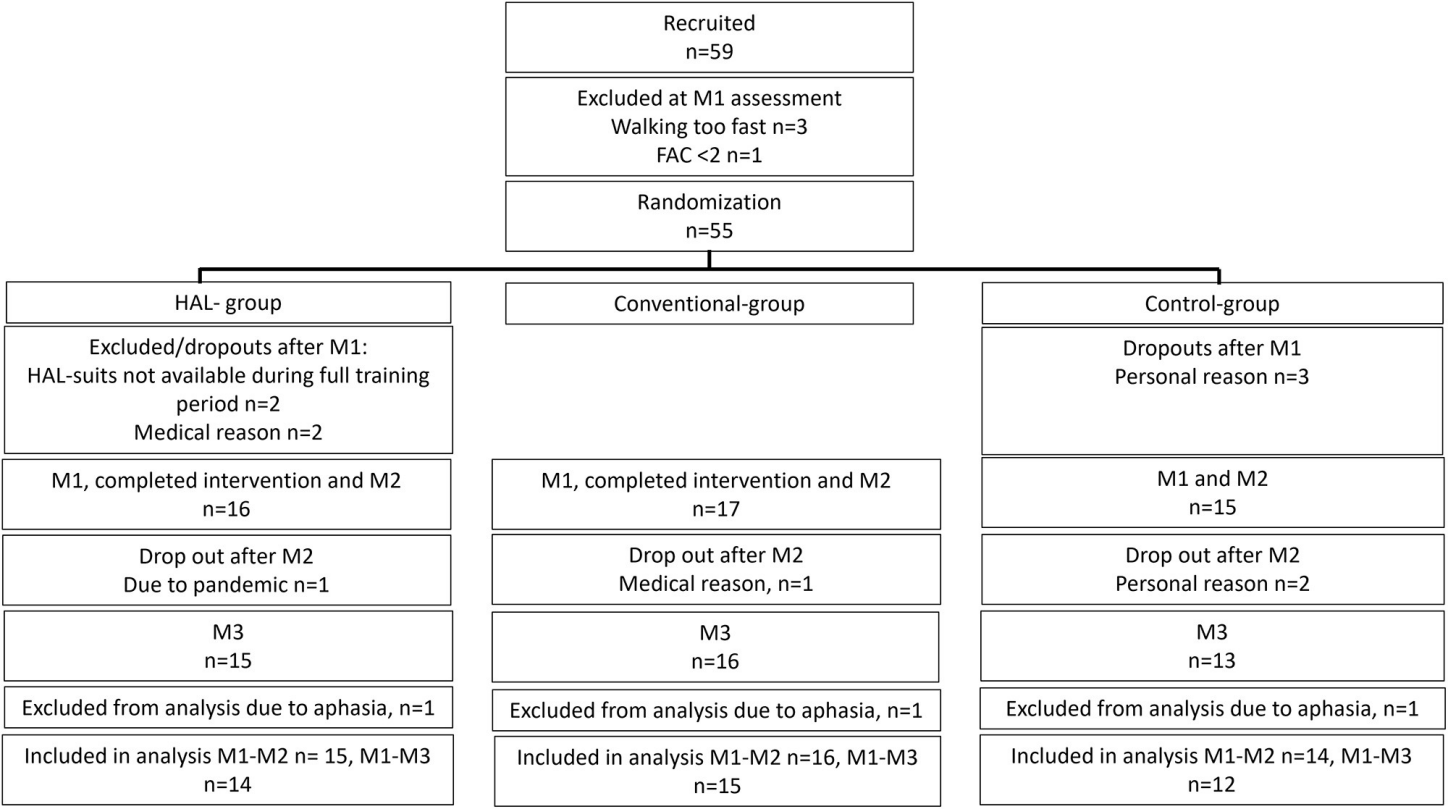
Flow chart of included participants. M1 = Baseline assessment, M2 = 6-week assessment, M3 = 6-month assessment.

A total of 45 participants were included in this study and a flow chart of the inclusion is presented in Fig 1.

Descriptive data of the participants are presented in Table 1. Statistically significant correlations were seen between the following descriptive data and outcome measures: Gender and DEX-SO (*r* = -.471, *p* = .002), BI (*r* = .412, *p* = .005), BBS (*r* = .39, *p* = .008) and SIS-ADL (*r* = .295, *p* = .049). NIHSS and DEX-SO (*r* = .489, *p* = .001), BI (*r* = .340, *p* = .022) and BBS (*r* = -.05, *p* = .001). Age and FAC (*r* = -.304, *p* = .042) and SIS-ADL (*r* = -.310, *p* = .038). No significant correlations were seen between MoCA Vis/Ex and the descriptive data (p≥.182).

**Table 1:** Baseline characteristics.

	All participants (n = 45)	HAL-group (n = 15)	Conventional group (n = 16)	Control group (n = 14)
**Age (years), m (IQR)**	64 (58–68)	64 (59–66)	66 (58–68)	61 (47–69)
**Time to inclusion (months), m (IQR)**	29 (19–47)	21 (17–41)	39 (24–66)	32 (16–67)
**Gender male, n (%)**	32 (71)	10 (67)	10 (62.5)	12 (86)
**Diagnosis, n (%)**				
• **Hemorrhagic**	14.(31)	3 (20)	7 (44)	4 (29)
• **Ischemic**	30 (67)	11 (73)	9 (56)	10 (71)
• **Both**	1 (2)	1 (7)	0 (0)	0 (0)
**Hemiparesis left, n (%)**	32 (71)	10 (67)	13 (81)	9 (64)
**Education level, n (%)**				
• **Elementary school**	9 (20.5)	3 (20)	4 (27)	2 (14)
• **High school**	13 (29.5)	6 (40)	2 (13)	5 (36)
• **University**	22 (50)	6 (40)	9 (60)	7 (50)
**NIHSS, m (IQR)**	7 (5–9)	7 (3–8)	7.5 (4–10)	8 (5–10)
**MoCA, m (IQR)**	24 (21–26) *	23 (22–26) ^	24 (20–27) ¨	23 (19–27) ¤
**Fugl-Meyer LE, m (IQR)**	61 (54–68)	64 (55–67)	60 (54–70)	59 (50–68)
**6-minute walk test, m (IQR)**	93 (38–135)	67 (30–123)	99 (34–120)	108 (50–168)
**10-meter walk test, m (IQR)**	0.31 (0.16–0.41)	0.21 (0.12–0.41)	0.32 (0.17–0.39)	0.33 (0.16–0.52)
**FAC, n (%)**				
• **FAC 2**	14 (31.1)	7 (46.7)	4 (25)	3 (21)
• **FAC 3**	10 (22.2)	1 (6.7)	4 (25)	5 (36)
• **FAC 4**	21 (46.7)	7 (46.7)	8 (50)	6 (43)
**Berg Balance Scale, m (IQR)**	37 (25–44)	37 (24–45)	40 (23–45)	34 (25–42)
**Barthel Index, m (IQR)**	85 (70–90)	80 (70–90)	85 (65–99)	82.5 (74–88)
**SIS-Mob, m (IQR)**	67 (53–81)	61 (53–72)	68 (51–86)	67 (63–79)
**SIS-ADL, m (IQR)**	55 (48–69)	50 (48–75)	56 (48–74)	55 (40–68)

Values shown in median (m) and Interquartile range (IQR), or frequency (n) and percent (%). NIHSS = National Institute of Health Stroke Scale, MoCA = Montreal Cognitive Assessment, Fugl-Meyer LE = Fugl-Meyer Lower Extremity. FAC = Functional Ambulation Classification, SIS-Mob = Stroke Impairment Scale domain 6 mobility, SIS-ADL = Stroke Impairment Scale domain 5 activities of daily living. Data missing due to aphasia (≥1 item missing):

* n = 38,

^ n = 13,

¨ n = 14, ¤ n = 11.

### Associations between baseline visuospatial and executive function and activity performance

In the analysis of the assessed baseline variables, as presented in Table 2, moderate to strong positive correlations were seen between MoCA Vis/Ex and 6MWT, 10MWT, BBS and FAC, with the strongest correlation to BBS. Among the self-perceived variables, moderate to strong correlations were seen between MoCA Vis/Ex and BI, SIS-Mob and SIS-ADL, with the strongest correlation to BI. There were no significant correlations between the rated DEX-SO and any of the assessed or self-perceived variables of activity performance (Table 2).

**Table 2:** Correlation matrix including baseline visuospatial and executive function and baseline measures of assessed and self-perceived activity performance.

	MoCA Vis/Ex (n = 45)		DEX-SO (n = 40)	
	Corr. (*r*)	Sig. (*p*)	Corr. (*r*)	Sig. (*p*)
**6 Minute Walk Test**	.358*	.016	.071	.665
**10 Meter Walk Test**	.414*	.005	-.011	.947
**Berg Balance Scale**	.687*	.000	-.216	.181
**FAC**	.491*	.001	-.145	.370
**Barthel Index**	.528*	.000	-.143	.378
**SIS-Mob**	.498*	.001	-.094	.563
**SIS-ADL**	.341*	.022	-.143	.377

MoCA Vis/Ex = Montreal Cognitive Assessment Visuospatial/Executive domain, DEX-SO = Dysexecutive Questionnaire rated by significant other, FAC = Functional Ambulation Classification, SIS-Mob = Stroke Impairment Scale domain 6 mobility, SIS-ADL = Stroke Impairment Scale domain 5 activities of daily living.

* = Significance at adjusted alpha-level *p* < .025.

Results from the multivariable linear regression analyses are presented in Table 3. In the ordinal regression analysis, MoCA Vis/Ex was found to explain 33% of the variance of the FAC (*p* = .005, Pseudo R2 Nagelkerke = .326).

**Table 3:** Results of the multivariable linear regression analyses, presented as coefficient of determination (*r*^*2*^) for baseline MoCA Vis/Ex along with included covariates in the model (NIHSS, age, gender) and baseline measures of activity performance.

	Model			MoCA Vis/Ex contribution to model	
	r^2^	Sig. (*p*)	Covariates of significance	*r* ^2^	Sig. (*p*)
**6-minute walk test**	.145	.006	MoCA Vis /Ex	.145	.006
**10-meter walk test**	.160	.004	MoCA Vis/Ex	.160	.004
**Berg balance scale**	.536	.000	MoCA Vis/Ex, NIHSS	.329	.000
**Barthel index**	.355	.025	MoCA Vis/Ex, age, gender	.098	.010
**SIS-Mob**	.222	.001	MoCA Vis/Ex	.222	.001
**SIS-ADL**	.138	.045	Age, gender	.055	.068

Note: Presented are values for MoCA Vis/Ex, when adjusted for significant covariates, along with significant models.

SIS-Mob = Stroke Impairment Scale domain 6 mobility, SIS-ADL = Stroke Impairment Scale domain 5 activities of daily living. MoCA Vis/Ex = MoCA Visuospatial/executive domain, NIHSS = National Institute of Health Stroke Scale. N/A: Nonsignificant in the multivariable linear regression analysis.

### Association between visuospatial and executive function and mobility outcome after intervention

**Fig 2:**
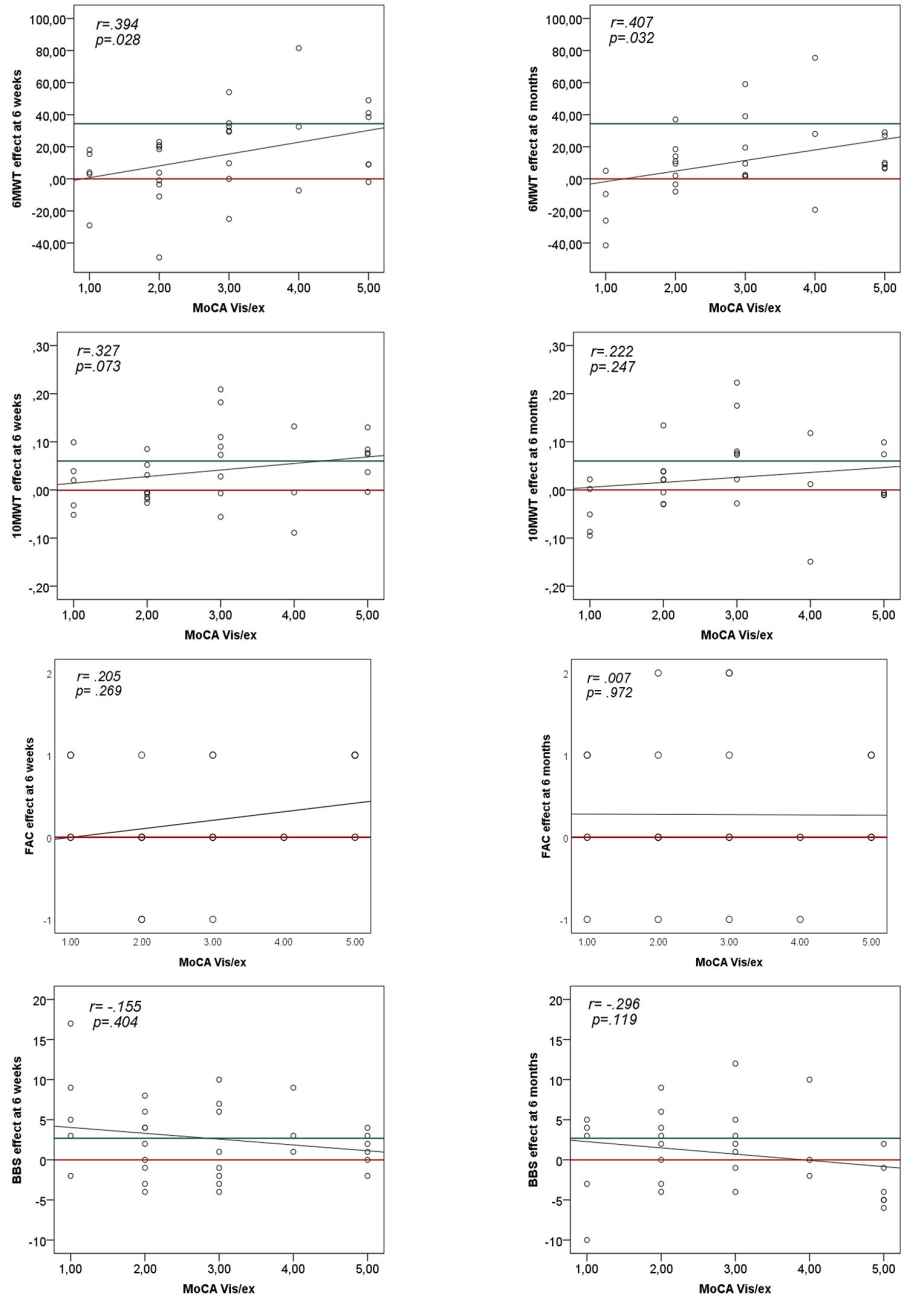
Scatterplots of the association between MoCA Vis/Ex and the effect on mobility outcome at 6 weeks (ΔM2-M1) and 6 months (ΔM3-M1), for the intervention groups together. Red line = 0. Green line = Minimally Clinical Important Difference (MCID). 6MWT = 6-minute walk test, 10MWT = 10-meter walk test, BBS = Berg Balance Scale. Adjusted alpha-level = *p* < .004.

Descriptive data on mobility outcome at 6 weeks and 6 months, can be seen in Table 4, as well as scatterplots visualizing the association in Fig 2.

**Table 4:** The effect on mobility outcome at 6 weeks (ΔM2-M1) and 6 months (ΔM3-M1).

	Intervention groups (HAL + conventional) (n = 31)	HAL-group (n = 15)	Conventional group (n = 16)	Control group (n = 14)
**6 MWT (meters)**				
**• ΔM2-M1**	16 (-1–33)	10 (-2–29)	18 (3–38)	-2 (-13–7)
**• ΔM3-M1**	9 (2–25)	14 (4–29)*	7 (-10–11)^	-4 (-43–8)¨
**10 MWT (m/s)**				
**• ΔM2-M1**	.03 (-.01 – .09)	.02 (-.02 –.07)	.06 (-.01 –.10)	.01 (-.02 -.09)
**• ΔM3-M1**	.02 (-.02 – .08)	.02 (-.01 –.08)*	.00 (-.03 –.04)^	-.01 (-.06 -.05)¨
**FAC (points)**				
**• ΔM2-M1**	0 (0–1)	0 (0–1)	0 (0–0.75)	0 (0–0)
**• ΔM3-M1**	0 (0–1)	0 (0–1.25)*	0 (0–1)^	0 (-.75–1)¨
**BBS (points)**				
**• ΔM2-M1**	2 (-1–6)	1 (-2–7)	3 (-1–6)	3 (2–4)
**• ΔM3-M1**	1 (-4–4)	1 (-4–5)*	2 (-3–3)^	0 (-3–5)¨

Note: Values are displayed as median and interquartile range (IQR).

M1 = Baseline assessment, M2 = 6-week assessment, M3 = 6 months assessment, 6MWT = 6-minute walk test, 10MWT = 10-meter walk test, FAC = Functional Ambulation Classification, BBS = Berg balance scale.

Number due to dropout:

* n = 14,

^ n = 15,

¨n = 12

As illustrated in the scatterplots in Fig 2, a higher MoCA Vis/Ex score (3–5) indicated larger improvements on the 6MWT, while a lower MoCA Vis/Ex score (1–2) indicated a decline or limited improvement both at 6 weeks and 6 months. Furthermore, participants with the lowest score on MoCA Vis/Ex (1), mainly presented a decline at 6 months in both 6MWT and 10MWT. For BBS, participants with the highest MoCA Vis/Ex score (5) showed limited improvements at 6 weeks and a decline at 6 months, while participants with 1 point on MoCA Vis/Ex generally improved both at 6 weeks and 6 months, though not significant in the correlation analysis (Fig 2). No associations were presented between MoCA Vis/Ex and effect on FAC at 6 weeks or 6 months (Fig 2).

Further, when observing the association between the DEX-SO and effects on mobility outcome at 6 weeks and 6 months, only the 10MWT at 6 weeks presented a significant correlation (Table 5). In the linear regression analysis, DEX-SO explained the variance in 10MWT by 23% (*r*^*2*^ = .232, p = .006), adjusted for gender and NIHSS. As seen in Table 5, no significant correlations were seen between DEX-SO and BBS, FAC or 6MWT at 6 weeks and 6 months, nor to effect in 10MWT at 6 months.

**Table 5:** Associations (*r*) between Dysexecutive function rated by significant other (DEX-SO) and effects on mobility outcomes after intervention, presented for the intervention groups.

	Intervention groups (HAL+Conventional) (n = 28)	HAL-group (n = 14)	Conventional group (n = 14)
	Corr. (*r*)	Corr. (*r*)	Corr. (*r*)
**6MWT ΔM2-M1**	-.443	-.391	-.341
**6MWT ΔM3-M1**	-.230	-.063	-.301
**10MWT, ΔM2-M1**	-.537*		
**10MWT, ΔM3-M1**	-.133		
**FAC ΔM2-M1**	-.126		
**FAC ΔM3-M1**	-.061		
**BBS ΔM2-M1**	.030		
**BBS ΔM3-M1**	.122		

M1 = Baseline assessment, M2 = 6-week assessment, M3 = 6 months assessment, 6MWT = 6-minute walk test, 10MWT = 10-meter walk test, FAC = Functional Ambulation Classification, BBS = Berg balance scale.

* = Significance at adjusted alpha-level *p* < .004.

N/A = Outcome measures not included in the groupwise analysis.

### Associations between visuospatial and executive functions and achieved walking distance after robotic or conventional gait training

**Fig 3:**
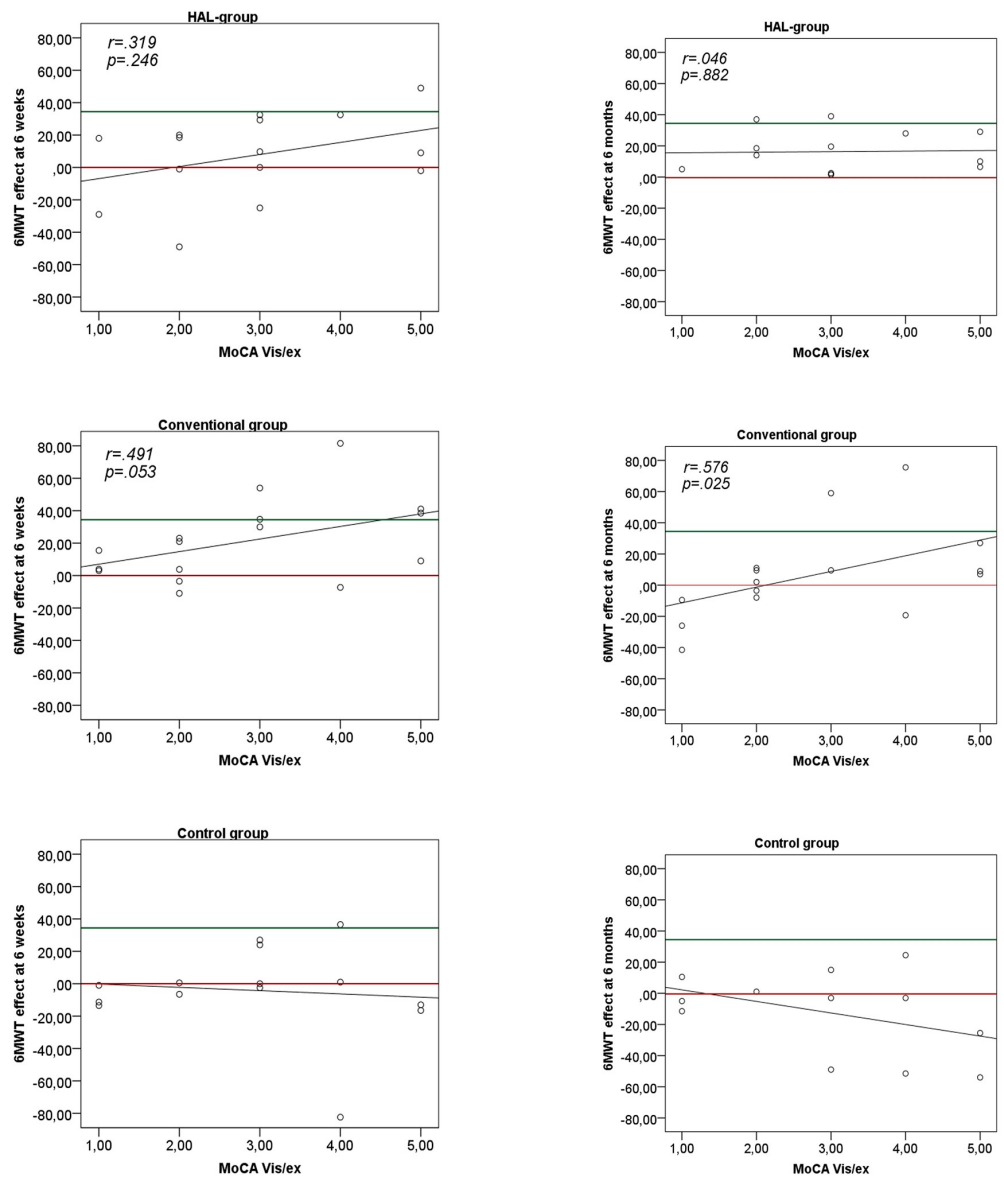
Scatterplots of the association between Montreal Cognitive Assessment visuospatial/executive domain (MoCA Vis/Ex) and effect on 6-minute walk test (6MWT) at 6 weeks and 6 months, for all groups separately. Correlations by Spearman’s rho = *r*. Red line = 0. Green line = Minimally Clinical Important Difference (MCID). Adjusted alpha-level = *p*≤ .006.

The association between MoCA Vis/Ex and the effect on 6MWT at 6 weeks and 6 months according to group allocation, are presented in Fig 3. In the HAL-group, improvement at 6 weeks and 6 months was not associated with the MoCA Vis/Ex score. In the conventional group, on the other hand, a significant association between improvements in 6MWT and MoCA Vis/Ex was found at 6 months and was also indicated at 6 weeks according to the scatter plot in Fig 3 (although not statistically significant p = 0.053). At both time points participants in the conventional group, with a higher score on the MoCa Vis/Ex (3–5) showed a greater improvement compared to those with a lower score (1–2). Moreover, none of the participants with MoCA Vis/Ex 1–2 acquired improvement above the MCID on 6MWT at 6 months. No significant associations with the DEX-SO were found (Table 5).

In the linear regression analyses exploring the association between the MoCA Vis/Ex score when dichotomized (1-2/3-5 points) and the effect on 6MWT at 6 weeks and 6 months, no significant associations were found in the HAL group at 6 weeks (*r*^*2*^ = 0.131 *p* = 0.184) or 6 months (*r*^*2*^ = 0.014 *p* = 0.698). In the Conventional group however MoCA Vis/Ex Dich explained 34% of the variance in the effect on 6MWT at 6 weeks (*r*^*2*^ = 0.344 *p* = 0.017) and 31% at 6 months (*r*^*2*^ = 0.308 *p* = 0.032).

## Discussion

The current study indicates that associations between visuospatial and executive function and activity performance, previously demonstrated in the early stage after stroke, remain long-term. Notably, visuospatial/executive function was associated with the effect of conventional gait training both at 6 weeks and 6 months, where a higher level of visuospatial/executive function indicated a larger improvement. In the HAL-group no such association was found, indicating improvements all over the spectrum of assessed visuospatial/executive function. Executive function rated by significant others did not present any notable associations.

Cognitive impairments in general have been associated with activity performance in several studies in the acute and subacute stages [10–12, 14, 16]. The current study suggests a continuance of this association into the later stages (1–10 years) after stroke. It seems likely that not only cognition in general, but visuospatial and executive function in particular, can be associated with activity performance [13–20]. This hypothesis is supported by the results of the current analysis, showing moderate to strong associations with the assessed visuospatial/executive function. The strongest association was seen between assessed visuospatial/executive function and balance, possibly reflecting the more complex and multi-task components included in the balance test, in contrast to the single task walking tests.

Visuospatial and executive function have been positively associated with long-term improvement in balance, up to one year after stroke [18]. The current study supports this relationship after more than one year post stroke. On the other hand, results of the current study showed that those with a higher level of visuospatial/executive function generally showed less improvement in balance function. This is considered unexpected, as balance may potentially affect improvements in walking ability [50]. A plausible explanation may be that those with a higher level of visuospatial/executive function also had a higher baseline balance function, leaving less margins of improvement, especially as the gait training did not include specific balance-training. However, these results must be interpreted with caution since no statistical significance was reached. Nevertheless, the current analysis implies future research focusing on how specific aspects of visuospatial and executive function can predict activity performance long-term, to further identify factors able to predict poor activity performance.

In the analysis of the association between visuospatial/executive function and the effect of the gait interventions, a larger impact on walking distance and -speed was seen at 6 months, compared to 6 weeks. These results indicate that generalization of training results to everyday activities can be negatively affected by visuospatial and executive impairments impacting on problem-solving, interpretation of the body and environment, processing of feedback, self-regulation and initiative [8, 9]. One example is the aspect of visuospatial impairments that may affect orientation to place, leading to an insecurity in walking outdoors and thus, prohibiting gait training in the local environment. Additionally, limitations in initiation and planning of activities related to executive function, may contribute to a sedentary lifestyle, limited ability to participate in rehabilitation interventions and to sustain achieved improvements [17].

When comparing the groups separately, the HAL-group gained improvements in walking distance at 6 months all over the spectrum of assessed visuospatial/executive function. In contrast, the conventional group showed that visuospatial/executive function was associated with the outcome of the gait intervention, where those with a lower score generally acquired small or no improvements at 6 weeks and 6 months. Based on these results, it is plausible that HAL-training may be more suitable than conventional gait training for participants with more severe visuospatial/executive impairments. On the other hand, for those with more preserved visuospatial/executive function, conventional gait training seems to have more beneficial effects in terms of MCID, indicating cognitive capacity enabling engagement in rehabilitation interventions and generalization to everyday activities, promoting long-term sustainability of achieved improvements.

Considering the association between level of visuospatial/executive impairments and limitations in walking, as shown in the baseline analysis, finding effective gait training interventions for people with visuospatial and executive impairments is crucial. Since the HAL-training is both task-specific and repetitive, HAL-training may have the potential to improve walking ability among patients with more severe visuospatial/executive impairments. Theories of neuroplasticity in the central nervous system support this hypothesis, where a reorganization of recruitment patterns and -areas in the brain can be seen as a response to altered input and demands, both in healthy subjects and in a stroke population [51]. There is increasing evidence that task-specific training post stroke is associated with such changes in the brain, leading to structural and functional changes affecting activity performance [51]. However, methods must be developed to facilitate generalization to activity performance in this patient group.

Although participants with visuospatial/executive impairments gained improvement at 6 months in the HAL-group, only a few acquired effects exceeding the MCID. These results can though be set in comparison to the control group, where a decline in walking distance at 6 months was seen. The results further imply that even with a well preserved visuospatial/executive function, a decline in activity performance may be seen long-term. This highlights the need of individualized interventions to preserve activity performance over time, even long-term after stroke when rehabilitation interventions usually are sparse [5]. Further, it is crucial to optimize the interventions by taking both cognitive and physical aspects into consideration when designing and setting goals for a rehabilitation intervention. Results from the current analysis indicate that robotic gait training when combined with conventional training as performed in the current study, has the potential of sustaining and improving walking capacity among persons with visuospatial/executive impairments, in a time period when a decline is otherwise commonly present. Results of the current study can be used to design future larger studies evaluating the effect of robotic gait training among persons with severe visuospatial and executive impairments.

Results from this study may not be generalized to the whole stroke population but targets a younger subgroup living with moderately severe impairments and limitations in walking long-term after stroke.

The measures used for evaluating the visuospatial and executive function were MoCA Vis/Ex as a clinical assessment of visuospatial and executive function, as well as the DEX-SO for the perceived everyday impairments related to executive function. The MoCA is commonly used in clinical contexts and assesses multiple domains of cognition, enabling the use of sub scores for specific domains [52]. The current assessment with MoCA Vis/Ex, were easy to administer and proven reliable and valid for this group of patients. However, in future research, assessments made by a neuropsychologist targeting multiple aspects of executive and visuospatial function could be used to deepen the analyzes of which specific executive impairments affect long-term walking ability the most and increase the robustness of the results. The DEX-SO did not show similar associations to activity performance, possibly related to the fact that the DEX-SO includes additional aspects of executive function compared to the MoCA Vis/Ex. These aspects may not be associated with motor performance (e.g., social, emotional and behavioral).

In the data analysis, action was taken to prevent errors due to multiple comparisons (Type-1 error) by applying the adjusted alpha level. On the other hand, the relatively small number of participants in the analysis of the association between visuospatial/executive function and the effect of gait training, can limit the ability of reaching statistical significance, as a Type-2 error. Altogether, the few significant associations presented can be assumed robust, results not reaching statistical significance can still be discussed as they might show tendencies of interest for future research. In the analysis of the groups separately, the results must be considered preliminary, due to the low number of participants, where changes or drop-outs of a few participants may have affected the results.

As described earlier, it is of great importance to investigate the effect of cognitive impairments on rehabilitation outcome, since it has been associated with poorer activity performance, both short- and long term after stroke [12, 13, 20]. It would also be of great value to investigate potential effects of combining training of executive functions with motor training on long-term walking ability among patients with moderate to severe stroke.

## Conclusion

The results of the current study indicate that the previously shown associations between visuospatial and executive function, and activity performance, in the early stage after stroke, remain long-term. Additionally, visuospatial/executive function was associated with the effect of conventional gait training both at 6 weeks and 6 months, where a higher level of visuospatial/executive function indicated a larger improvement. In the HAL-group no such association was found, indicating improvements all over the spectrum of assessed visuospatial/executive function. These results imply that HAL-training may be suitable for persons with more severe visuospatial/executive impairments who may not benefit from conventional gait and mobility training. However, additional studies are needed to explore this association further. Also, future research is suggested to evaluate specific aspects of visuospatial and executive functions, measured by a larger battery of cognitive assessment tools, to identify aspects especially important in predicting mobility outcome after an intervention. This can improve the accuracy in individualizing gait interventions to achieve the largest improvements also among persons with more severe cognitive impairments.

## Supporting information

S1 File HAL-project plan for ethical approval. (DOCX)

S1 Checklist CONSORT checklist. (PDF)

S1 Data (XLS)

## References

[1] Collaborators GDaH. Global, regional, and national disability-adjusted life-years (DALYs) for 359 diseases and injuries and healthy life expectancy (HALE) for 195 countries and territories, 1990–2017: a systematic analysis for the Global Burden of Disease Study 2017. Lancet. 2018;392(10159):1859–922. doi: 10.1016/S0140-6736(18)32335-3 30415748 PMC6252083

[2] Stokes M. Physical management in neurological rehabilitation. 2nd ed. Edinburgh; New York: Elsevier Mosby; 2004.

[3] Lee KB, Lim SH, Kim KH, Kim KJ, Kim YR, Chang WN, et al. Six-month functional recovery of stroke patients: a multi-time-point study. Int J Rehabil Res. 2015;38(2):173–80. doi: 10.1097/MRR.0000000000000108 25603539 PMC4415968

[4] Branco JP, Oliveira S, Sargento-Freitas J, Laíns J, Pinheiro J. Assessing functional recovery in the first six months after acute ischemic stroke: a prospective, observational study. Eur J Phys Rehabil Med. 2019;55(1):1–7. doi: 10.23736/S1973-9087.18.05161-4 29764094

[5] Teasell R, Mehta S, Pereira S, McIntyre A, Janzen S, Allen L, et al. Time to rethink long-term rehabilitation management of stroke patients. Top Stroke Rehabil. 2012;19(6):457–62. doi: 10.1310/tsr1906-457 23192711

[6] Donovan NJ, Kendall DL, Heaton SC, Kwon S, Velozo CA, Duncan PW. Conceptualizing functional cognition in stroke. Neurorehabil Neural Repair. 2008;22(2):122–35. doi: 10.1177/1545968307306239 17761809

[7] Jokinen H, Melkas S, Ylikoski R, Pohjasvaara T, Kaste M, Erkinjuntti T, et al. Post-stroke cognitive impairment is common even after successful clinical recovery. Eur J Neurol. 2015;22(9):1288–94. doi: 10.1111/ene.12743 26040251

[8] Anderson V, Jacobs R, Anderson PJ. Executive functions and the frontal lobes: A lifespan perspective. New York, London: Taylor & Francis; 2008.

[9] Cimadevilla JM, Piccardi L. Spatial skills. Handb Clin Neurol. 2020;175:65–79. doi: 10.1016/B978-0-444-64123-6.00006-0 33008544

[10] Ursin MH, Bergland A, Fure B, Thommessen B, Hagberg G, Øksengård AR, et al. Gait and balance one year after stroke; relationships with lesion side, subtypes of cognitive impairment and neuroimaging findings-a longitudinal, cohort study. Physiotherapy. 2019;105(2):254–61. doi: 10.1016/j.physio.2018.07.007 30340837

[11] Leniak M, Bak T, Czepiel W, Seniów J, Cz onkowska A. Frequency and Prognostic Value of Cognitive Disorders in Stroke Patients. Dement Geriatr Cogn Disord. 2008;26(4):356–63. doi: 10.1159/000162262 18852488

[12] Ginex V, Vanacore N, Lacorte E, Sozzi M, Pisani L, Corbo M, et al. General cognition predicts post-stroke recovery defined through minimal clinically important difference (MCID): a cohort study in an Italian rehabilitation clinic. Eur J Phys Rehabil Med. 2015;51(5):597–606. 25375185

[13] Liu-Ambrose T, Pang MY, Eng JJ. Executive function is independently associated with performances of balance and mobility in community-dwelling older adults after mild stroke: implications for falls prevention. Cerebrovasc Dis. 2007;23(2–3):203–10. doi: 10.1159/000097642 17143004 PMC4492718

[14] Nys GMS, van Zandvoort MJE, de Kort PLM, van der Worp HB, Jansen BPW, Algra A, et al. The prognostic value of domain-specific cognitive abilities in acute first-ever stroke. Neurology. 2005;64(5):821–7. doi: 10.1212/01.WNL.0000152984.28420.5A 15753416

[15] Pohjasvaara T, Leskelä M, Vataja R, Kalska H, Ylikoski R, Hietanen M, et al. Post‐stroke depression, executive dysfunction and functional outcome. Eur J Neurol. 2002;9(3):269–75. doi: 10.1046/j.1468-1331.2002.00396.x 11985635

[16] Mok VCT, Wong A, Lam WWM, Fan YH, Tang WK, Kwok T, et al. Cognitive impairment and functional outcome after stroke associated with small vessel disease. J Neurol Neurosurg Psychiatry 2004;75(4):560–6. doi: 10.1136/jnnp.2003.015107 15026497 PMC1739014

[17] Skidmore ER, Whyte EM, Holm MB, Becker JT, Butters MA, Dew MA, et al. Cognitive and Affective Predictors of Rehabilitation Participation After Stroke. Arch Phys Med Rehabil. 2010;91(2):203–7. doi: 10.1016/j.apmr.2009.10.026 20159122 PMC2824912

[18] Påhlman U, Gutiérrez-pérez C, Sävborg M, Knopp E, Tarkowski E. Cognitive function and improvement of balance after stroke in elderly people: the Gothenburg Cognitive Stroke Study in the Elderly. Disabil Rehabil. 2011;33(21–22):1952–62. doi: 10.3109/09638288.2011.553703 21306194

[19] Barrett AM, Muzaffar T. Spatial cognitive rehabilitation and motor recovery after stroke. Curr Opin Neurol. 2014;27(6):653–8. doi: 10.1097/WCO.0000000000000148 25364954 PMC4455599

[20] Oh-Park M, Hung C, Chen P, Barrett AM. Severity of spatial neglect during acute inpatient rehabilitation predicts community mobility after stroke. PM R. 2014;6(8):716–22. doi: 10.1016/j.pmrj.2014.01.002 24412266 PMC4090300

[21] Kwakkel G, Kollen B, Lindeman E. Understanding the pattern of functional recovery after stroke: facts and theories. Restor Neurol Neurosci. 2004;22(3–5):281–99. 15502272

[22] Bang D-H, Shin W-S. Effects of robot-assisted gait training on spatiotemporal gait parameters and balance in patients with chronic stroke: A randomized controlled pilot trial. NeuroRehabilitation. 2016;38(4):343–9. doi: 10.3233/NRE-161325 27061162

[23] De Luca A, Vernetti H, Capra C, Pisu I, Cassiano C, Barone L, et al. Recovery and compensation after robotic assisted gait training in chronic stroke survivors. Disabil Rehabil Assit Technol. 2018;14(8):1–13. doi: 10.1080/17483107.2018.1466926 29741134

[24] Wallard L, Dietrich G, Kerlirzin Y, Bredin J. Effects of robotic gait rehabilitation on biomechanical parameters in the chronic hemiplegic patients. Neurophysiol Clin. 2015;45(3):215–9. doi: 10.1016/j.neucli.2015.03.002 26381192

[25] Mehrholz J, Thomas S, Werner C, Kugler J, Pohl M, Elsner B. Electromechanical-assisted training for walking after stroke. Cochrane Database Syst Rev. 2017;5(5):Cd006185. doi: 10.1002/14651858.CD006185.pub4 28488268 PMC6481755

[26] Tedla JS, Dixit S, Gular K, Abohashrh M. Robotic-Assisted Gait Training Effect on Function and Gait Speed in Subacute and Chronic Stroke Population: A Systematic Review and Meta-Analysis of Randomized Controlled Trials. Eur Neurol. 2019;81(3–4):103–11. doi: 10.1159/000500747 31167193

[27] Bruni MF, Melegari C, De Cola MC, Bramanti A, Bramanti P, Calabrò RS. What does best evidence tell us about robotic gait rehabilitation in stroke patients: A systematic review and meta-analysis. J Clin Neurosci. 2018;48:11–7. doi: 10.1016/j.jocn.2017.10.048 29208476

[28] Tanaka H, Nankaku M, Nishikawa T, Hosoe T, Yonezawa H, Mori H, et al. Spatiotemporal gait characteristic changes with gait training using the hybrid assistive limb for chronic stroke patients. Gait Posture. 2019;71:205–10. doi: 10.1016/j.gaitpost.2019.05.003 31078010

[29] Palmcrantz S, Wall A, Vreede KS, Lindberg P, Danielsson A, Sunnerhagen KS, et al. Impact of Intensive Gait Training With and Without Electromechanical Assistance in the Chronic Phase After Stroke-A Multi-Arm Randomized Controlled Trial With a 6 and 12 Months Follow Up. Front. Neurosci. 2021 April 15:660726. doi: 10.3389/fnins.2021.660726 33967683 PMC8100236

[30] Wall A, Borg J, Palmcrantz S. Clinical application of the Hybrid Assistive Limb (HAL) for gait training-a systematic review. Front Syst Neurosci. 2015;9:48. doi: 10.3389/fnsys.2015.00048 25859191 PMC4373251

[31] Nasreddine ZS, Phillips NA, Bédirian Vr, Charbonneau S, Whitehead V, Collin I, et al. The Montreal Cognitive Assessment, MoCA: A Brief Screening Tool For Mild Cognitive Impairment. J Am Geriatr Soc. 2005;53(4):695–9. doi: 10.1111/j.1532-5415.2005.53221.x 15817019

[32] Bennett PC, Ong BEN, Ponsford J. Measuring executive dysfunction in an acute rehabilitation setting: Using the dysexecutive questionnaire (DEX). J Int Neuropsychol Soc. 2005;11(4):376–85. doi: 10.1017/s1355617705050423 16209417

[33] Pohl PS, Duncan PW, Perera S, Liu W, Lai SM, Studenski S, et al. Influence of stroke-related impairments on performance in 6-minute walk test. J Rehabil Res Dev. 2002;39(4):439–44. 17638141

[34] Tang A, Eng JJ, Rand D. Relationship Between Perceived and Measured Changes in Walking After Stroke. J Neurol Phys Ther. 2012;36(3):115–21. doi: 10.1097/NPT.0b013e318262dbd0 22850336 PMC3501529

[35] Flansbjer UB, Holmbäck AM, Downham D, Patten C, Lexell J. Reliability of gait performance tests in men and women with hemiparesis after stroke. J Rehabil Med. 2005;37(2):75–82. doi: 10.1080/16501970410017215 15788341

[36] Perera S, Mody SH, Woodman RC, Studenski SA. Meaningful change and responsiveness in common physical performance measures in older adults. J Am Geriatr Soc. 2006;54(5):743–9. doi: 10.1111/j.1532-5415.2006.00701.x 16696738

[37] Alghadir AH, Al-Eisa ES, Anwer S, Sarkar B. Reliability, validity, and responsiveness of three scales for measuring balance in patients with chronic stroke. BMC Neurol. 2018;18(1):141–7. doi: 10.1186/s12883-018-1146-9 30213258 PMC6136166

[38] Mehrholz J, Wagner K, Rutte K, Meissner D, Pohl M. Predictive validity and responsiveness of the functional ambulation category in hemiparetic patients after stroke. Arch Phys Med Rehabil. 2007;88(10):1314–9. doi: 10.1016/j.apmr.2007.06.764 17908575

[39] Duncan PW, Wallace D, Lai SM, Johnson D, Embretson S, Laster LJ. The stroke impact scale version 2.0. Evaluation of reliability, validity, and sensitivity to change. Stroke. 1999 Oct;30(10):2131–40. doi: 10.1161/01.str.30.10.2131 10512918

[40] Mahoney FI, Barthel DW. Functional evaluation: the Barthel Index. Md State Med J. 1965 Feb;14:61–5. 14258950

[41] Sheridan PL, Solomont J, Kowall N, Hausdorff JM. Influence of Executive Function on Locomotor Function: Divided Attention Increases Gait Variability in Alzheimer’s Disease. J Am Geriatr Soc. 2003;51(11):1633–7. doi: 10.1046/j.1532-5415.2003.51516.x 14687395

[42] Fong KNK, Chan CCH, Au DKS. Relationship of motor and cognitive abilities to functional performance in stroke rehabilitation. Brain Inj. 2001;15(5):443–53. doi: 10.1080/02699050010005940 11350658

[43] IBM SPSS Statistics for Windows, Version 22.0. Armonk, NY: IBM Corp.; 2013. https://www.ibm.com/products/spss-statistics

[44] McGlothlin AE, Lewis RJ. Minimal Clinically Important Difference: Defining What Really Matters to Patients. JAMA. 2014;312(13):1342–3. doi: 10.1001/jama.2014.13128 25268441

[45] Cohen J. Statistical Power Analysis for the Behavioral Sciences. 2nd ed. New York: Academic Press; 2013.

[46] Yoav B, Yosef H. Controlling the False Discovery Rate: A Practical and Powerful Approach to Multiple Testing. J Royal Statist Soc, Series B. 1995;57(1):289–300.

[47] Norman G. Likert scales, levels of measurement and the “laws” of statistics. Adv Health Sci Educ Theory Pract. 2010;15(5):625–32. doi: 10.1007/s10459-010-9222-y 20146096

[48] Björk J. Praktisk statistik för medicin och hälsa. 1. uppl. ed. Stockholm: Liber; 2011.

[49] Harrell FE. Regression modeling strategies: with applications to linear models, logistic and ordinal regression, and survival analysis. 2nd ed. Cham: Springer; 2015.

[50] Dobkin BHK, Nadeau SE, Behrman AL, Wu SS, Rose DK, Bowden M, et al. Prediction of responders for outcome measures of locomotor Experience Applied Post Stroke trial. J Rehabil Res Dev. 2014;51(1):39–50. doi: 10.1682/JRRD.2013.04.0080 24805892 PMC4374620

[51] Arya KN, Pandian S, Verma R, Garg R. Movement therapy induced neural reorganization and motor recovery in stroke: a review. J Bodyw Mov Ther. 2011;15(4):528–37. doi: 10.1016/j.jbmt.2011.01.023 21943628

[52] Toglia J, Fitzgerald KA, O’Dell MW, Mastrogiovanni AR, Lin CD. The Mini-Mental State Examination and Montreal Cognitive Assessment in persons with mild subacute stroke: relationship to functional outcome. Arch Phys Med Rehabil. 2011;92(5):792–8. doi: 10.1016/j.apmr.2010.12.034 21530727

