# Supplementary material for: The impact of visuospatial and executive function on activity performance and outcome after robotic or conventional gait training, long-term after stroke—as part of a randomized controlled trial
Source: PLoS One. 2023 Mar 9;18(3):e0281212. doi: 10.1371/journal.pone.0281212 (PMC9997896; doi:10.1371/journal.pone.0281212)
Supplement: S1 File — (DOCX) [file pone.0281212.s001.docx]

**New technology for individualised, intensive training of gait after stroke– phase III trials**

**Study II**

**Purpose and aims**

The overall purpose of this project is to establish the added value of training with the Hybrid Assistive Limb (HAL) exoskeleton system as part of regular rehabilitation intervention programs after stroke.

The main specific aims are

1. to demonstrate the effect of intensive training with HAL in comparison with intensive training without HAL and with standard care only, in a later phase (1-10 years) after stroke with regard to gait and cardio-vascular function, activity and participation;
2. to estimate health care utilisation and cost effectiveness of using the HAL during the first year after stroke.

**
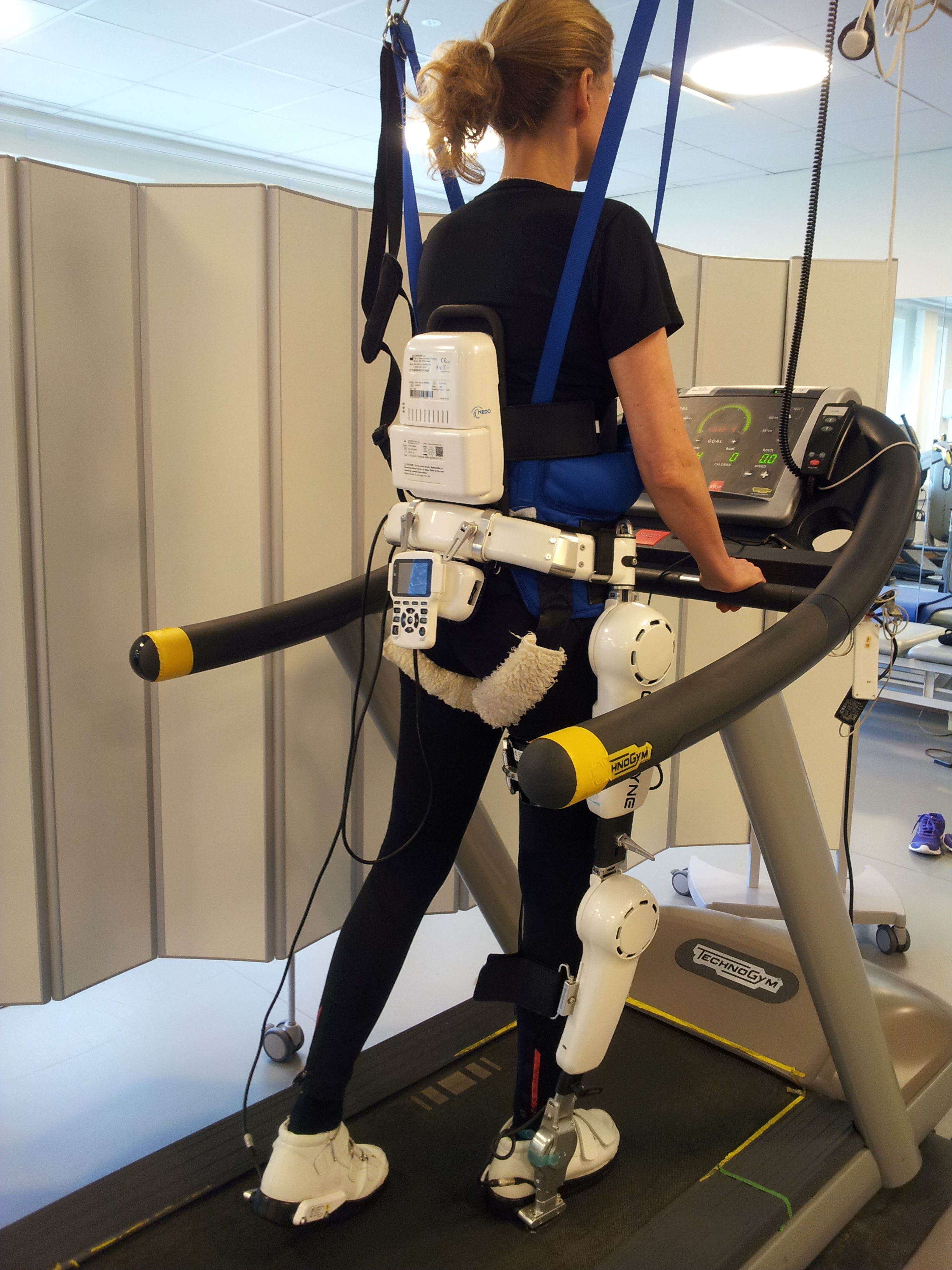
**

Figure 1. Illustratíon of HAL exoskeleton for gait training on treadmill with Body Weight Support.

***Survey of the field***

*Normal human gait* requires postural control, weight shifting and rhythmic and correct timing of muscle activity during repeated gait cycles and depends on the integrity of a complex interaction in sensory-motor neural networks at both spinal and supraspinal levels (Dietz et al 2002). Thus, gait may be impaired by a variety of brain disorders that cause a central paresis - including stroke, traumatic brain injury, cerebral palsy, multiple sclerosis.

*Stroke* is the third most common cause of death and the most common cause of acquired adult disability in developed countries (World Health Report 2003). Hemiparesis is the most common acute manifestation of stroke and often impacts on gait function (Jörgensen 1995). Depending on location and extent of the lesion and of restorative and compensatory mechanisms gait characteristics may vary between and within patients over time after stroke. Although performance is improved in most stroke survivors during the first months post stroke, one third or more will need assistance in walking and remains limited in community ambulation. Thus, independent gait remains a challenge after stroke (Dobkin 2005).

*Intensive, repetitive task specific training* may accelerate functional restitution after stroke and improve final motor outcomes (Langhorne et al 2009) but more effective training methods and a better understanding and prediction of the individual capacity and response to specific training paradigms remains a challenge **(**Bowden et al 2013).

*Conventional gait training* after stroke may include over ground walking with assistance and/or ambulatory devices or walking on a treadmill, with or without body weight support (BWS). Conventional training can be combined with electromechanical “gait machines”, which may allow more reproducible gait movements than manual movement support by a therapist.

*Gait machines* are often categorized as applying an end-effector principle or exoskeletons (Hesse etal 2010). End-effector machines use foot plates that move the feet in a controlled gait pattern and allow the operator to adjust aspects of locomotion, such as speed, stride length and step height. In contrast, exoskeletons such as Lokomat (Jezernik et al 2003) are attached to the patient and function as an external skeleton. Exoskeletons for lower extremities have joints matching the patient’s lower limb joints and motors that drive movements over these joints to assist leg movements.

*A recent Cochrane review* concluded that electromechanical-assisted gait training in combination with physiotherapy after stroke increased the odds of participants becoming independent in walking and most so when this is applied in the first three months after stroke in patients, who are not able to walk, and also concluded that further studies are needed with regard to the role of current types of electromechanical device (Merholz et al 2013). There is also a recognised need for new concepts and devices and their evaluation in clinical trials (Pennycott et al 2012).

*A key conceptual issue* relates to the importance of incorporating active participation in the training. This has been approached in several studies by comparing training by use of gait machines such as Lokomat or Gait Trainer only, with regular therapist training that allows more variation, or combinations of these where the patient is more active. Recently, an exoskeleton with a hybrid system that allows both an automatic and a voluntary mode of action to support training of gait, the Hybrid Assistive Limb system (HAL) has been developed and introduced in clinical trials (Kawamoto et al 2010; Kawamoto et al 2013;Nilsson et al 2014; Watanabe 2014). This exoskeleton provides support according to the patient’s condition by a control algorithm and supporting devices, where each joint (left and right hip and left and right knee) can be controlled separately.

*The HAL system* comprises two subsystems for (cybernic) voluntary control (CVC) and (cybernic) autonomous control (CAC) respectively. Both modes of action depend on the user’s intention in different ways. The CAC mode utilizes voluntary weight shift to initiate gait cycles and then provides predefined movements while gait in the CVC mode continuously use input from voluntarily activated gait muscles to provide support by the exoskeleton. This is achieved by use of surface electromyography (EMG) signals (Kawamoto et al 2002) from lower extremity extensor and flexor muscles to initiate and adapt power output, which may then be modified by a therapist. The technology enables even weak EMG activity to be used to initiate and adjust the assistive torque in the CVC mode. Output is magnified and adjusted to the level of assistance needed over each hip and knee joint. A main controller of the system is used to control the power units, monitor the batteries, communicate with the system operator and modulate the assisting torque of each power unit. HAL is equipped with a sensing system receiving input also from potentiometers that are mounted on each joint and used as angular sensors to measure the joint angles, from force-pressure sensors in the shoes and from a gyro sensor and an acceleration sensor, which are mounted on the HAL body trunk, to measure posture (Kawamoto et al 2013)

*The CVC mode* allows the operator to adjust the degree of physical support for each joint and gradually reduce support as training progress. The EMG input, the adjusted torque limit and torque tuner for each joint and the adjusted assistance level for the flexor and extensor muscle groups respectively all together determine the power output. These settings can not be standardized but are individually adapted over time. Settings are modified by the therapist during the training session depending on the patient’s performance in order to achieve a gait pattern that is as close as possible to normal gait. If the subject is paralytic, as may be the case early after stroke, the CAC mode may be used. Gait is then initiated and sustained by the voluntary locomotor intention, based on output from force-pressure sensors in the shoes. In this mode, the exoskeleton will e.g. swing the left leg when enough weight is put on the right leg in stance phase.

*The feasibility, safety and potential functional benefits* of gait training with HAL after stroke have been demonstrated (Kawamoto et al 2013;, Watanabe et al 2014 ,Nilsson 2014 et al). Most trials have used the Functional Ambulation Category/FAC scale, which is a widely used and valid measure of independence in ambulation (Holden et al 1984) as primary outcome measure. Previous exploratory studies indicate that training with HAL may improve ambulation by one FAC category or more when compared with regular training programs, and this corresponds to a clinically meaningful effect. Further studies are needed to confirm these findings in phase III trials.

.

**Project description**

*Theory/Rationale*

*Current neurorehabilitation theories* are based on an understanding of how plasticity of the nervous system may be modulated by behaviour/activities and experiences to support recovery of function. Fundamental neurobiological components involved in this process include reversal of diaschisis, cell genesis, reorganisation and formation of neural pathways (Pekna et al 2012). These components vary over time and relate to spontaneous recovery (Duncan et al 1992), motor training, involvement of the contralateral hemisphere and remodelling of the corticospinal system.

*Identified key factors to optimize functional recovery after stroke are early onset, intensity and progression* of the rehabilitation interventions in order to drive beneficial neuroplasticity and thus to promote positive functional outcomes. In spite of a large number of randomized control trials (RCTs) in stroke rehabilitation have been published until now, little evidence from these has translated into clinical practice (Stinear et al 2013). Beneficial effects of intense and progressive training long-term after stroke have also been reported but further studies are needed to establish the effects (Veerbeek et al, 2014).

*The HAL system*, as reviewed above, offers a new method for intensive training of gait function that may start early after stroke also in patients with severe lower extremity paresis and there is evidence from phase I and phase II studies that this method adds value in terms of improved gait function after stroke. However, in order to introduce the method in clinical practice in neurorehabilitation, further studies are needed as outlined in this project.

*The expected recovery curve for gait function after stroke* needs careful consideration and most so when evaluating the effects during the early recovery phase after stroke (Kwakkel et al 2006). Clinically it remains challenging to determine who will respond well to gait interventions. To optimize rehabilitation efforts and goal setting simple and quick clinical measures for prediction of gait recovery are required. Identified markers of recovery of gait after stroke include initial balance test scores and walking speed and age (Dobkin, Duncan et al, 2014) while the role of initial range of movement and muscle strength and tone in the lower limb remains unclear. In the current project, we will investigate if active ankle range of movement, ankle strength or ankle spasticity are useful to predict gait recovery with HAL gait training. Spasticity at the ankle will be quantified using the NeuroFlexor method, which allows separate measurement of biomechanical (elasticity, viscosity) and neural (spasticity) components of passive resistance. The NeuroFlexor method has been shown to be valid and reliable for measurement of spasticity at the wrist (Lindberg et al, 2011) and validation of a foot module to measure ankle spasticity is in progress (Autumn 2014).

*Cardiovascular function* also needs careful consideration when designing interventions for motor impairments after stroke. Physical acitivity is a corner stone in the prevention and treatment of stroke (American Heart Association Circulation 2004). Treadmill training is considered an excellent training method and there is evidence that cardiorespiratory training including training of gait should be included in the rehabilitation program after stroke but further trials are needed to define the optimal exercise prescription and long-term benefits. (Brazzelli et al 2011). Thus, both potential limitations of the individual physical training capacity and the potentially beneficial impact that intensive gait training may have on cardiovascular function and secondary stroke prevention as well as on other than motor impairments, e.g. on cognition and mode must be considered. In this project, measures reflecting cardiovascular function will therefore be monitored.

***Methods and procedures***

Design: A multicentre, randomized, controlled phase III study will be conducted in 3 study sites in Sweden (Stockholm, Gothenburg and Umeå).

Study design and protocol will follow the CONSORT standards and are in accordance with the principles of the Declaration of Helsinki and Good Clinical Practice. The study protocol will be approved by the ethical committee in Stockholm and will be submitted for publication on *clinicaltrials.gov.*

Study Endpoints

The primary aim of the study is to compare the effectiveness of a) intensive training with HAL, b) intensive training without HAL and c) standard training, in a later phase (1-10 years) after stroke on independence in ambulation after intervention by comparing the Functional Ambulation Category in each group.

Secondary aims comprise the effectiveness of training with HAL on independence gait function and quality, cardiovascular function, activity of daily living and participation assessed at the end of intervention and at a 6 and 12 months follow-up. Effects on health care consumption during 12 months after the intervention will be explored and treatment related adverse events during the study will be documented. Further, associations between range of movement, strength and spasticity versus HAL mediated recovery will also be explored.

Setting: Setting: The study will be performed simultaneously at Danderyd University Hospital and Department of Clinical Sciences, Karolinska Institutet in Stockholm; Sahlgrenska University Hospital and University of Gothenburg in Gothenburg; Norrland University Hospital and Umeå University in Umeå.

*Recruitment of patients*

Eligible are persons, aged 18-70 years, recruited from out-patient rehabilitation units where they have passed a regular rehabilitation program due to stroke related hemiparesis/central paresis of the lower extremity (LE) after an ischemic or haemorrhagic first ever stroke (assessed by a stroke physician and verified by CT or MRI examination- this data will be collected from initial referral to the unit or in case of missing data, by contacting the physician or the medical records after approval by the study participant).

Inclusion criteria: 1-10 years since stroke onset; able to walk but not independently, i.e. need of manual support or close supervision due to lower extremity paresis, FAC score 2-3 or FAC 4 combined with gait speed <0.8m/s according to 10 meter walk test, which corresponds to limitations in community ambulation (Bowden et al 2008); ability to understand training instructions as well as written and oral study information and to express informed consent or by proxy; body size compatible with the HAL suit.

Exclusion criteria: contracture restricting gait movements at any lower limb joint; cardiovascular or other somatic condition incompatible with intensive gait training; severe, contagious infections (e.g. Methicillin Resistant Staphylococcus Aureus (MRSA) or Extended Spectrum Beta Lactamase bacteria).

*Randomisation*

Randomisation will be performed by a clinician, who is not otherwise involved in the study according to a block design procedure where patients are randomized into each group.

*Interventions*

To standardize the training procedure, training with HAL is performed on a treadmill and to enable body weight support. Body weight support is used to prevent falls and to unburden the weight of the suit (9 kg). The training program is performed by physiotherapists, trained in the HAL method and the study procedures.

The conventional gait training is performed according to current best evidence based practice and may include over ground walking with assistance and/or assistant devices as well as the use of a treadmill and body weight support and training of gait function in activities of daily living

Intensive gait training with HAL is performed 1 session/day, 3 days/week for 6 weeks and each session will not exceed 60 min of effective walking time with HAL. In addition, each session will include conventional gait training that will not exceed 30 min effective training time. At the end of the 6 weeks, the physiotherapist that has been engaged in the patient’s conventional training will perform 1-2 home visits to inform/educate the patient and those who are providing assistance to the patient in how the patient can make use of any gains in gait function during activities of daily living. The 1^st^ control group will receive conventional gait training performed 1 session/day, 3 days/week for 6 weeks that will not exceed 1h 30 min effective training time. The 2nd control group will not receive an intervention.

*Data collection*

All data collection will follow and be documented in individual Case Report Forms (CRF).

The HAL-training in Study I and II will documented in a standardized protocol, developed and tested in previous and ongoing studies. Conventional gait training in Study I will be documented based on data collected from medical records at the rehabilitation units.

Intensive gait training with and without HAL in Study II will be documented according to preformed forms. The participants in the 2d control group in who are not receiving an intervention, will document any scheduled training sessions in an web based application (app) or will be interviewed once a week according to a standardized protocol.

*Assessments*

Assessments will be made with valid instruments (not referenced in detail here) and performed once at baseline, immediately after the intervention period and at 6 and 12 months after the intervention period by a senior physician specialist physician, and by a blinded, experienced physiotherapist. The time points for the assessments are presented in the Table.

*Screening of function and disability:*

Data on Stroke type and localization will be collected from the medical records at the study sites. Body function will be assessed by use of the NIH Stroke Scale (NIHSS), mental function with Montreal Cognitive Assessment, executive function with the questionnaire Dysexecutive Questionnaire included in BADS (Behavioural Assessment of the Dysexecutive Syndrome), anxiety and depression with the Hospital Anxiety and Depression Scale and activity and participation by means of the Barthel Index and Modified Rankin Scale.

*Primary outcome measure*

The primary outcome will be the Functional Ambulation Categories (Holden 1984) that assesses activity in terms of independence in walking.

*Secondary outcome measures:*

Body function in terms of sensory function, pain and control of voluntary movement will be assessed with the Fugl Meyer Scale (FM-LE) and spasticity in the lower extremities with the Modified Ashworth Scale and the neuroflexor module and range of movement with the goniometer. Gait pattern function and walking will be assessed in laboratory gait analyses.

Activities and Participation in terms of walking will be tested with the 10 meter walk test, 2 and 6 minutes’ walk test combined with the Borg RPE scale for endurance, the accelerometer SenseWear Pro2 will be used to assess energy expenditure and physical activity (number of steps) in everyday life balance in activity with the Berg Balance Scale, and perceived functioning and disability with the Stroke Impact Scale (SIS).

Qualitative evaluation based on interviews will be performed in subgroups. Treatment related adverse events will be documented.

Data on registered health care and rehabilitation utilization during 12 months after the intervention period will be retrieved from the local County Councils at the study sites.

*Other assessments*

Resistance to passive stretch of lower leg muscles (spasticity) by use of new Neuroflexor/foot module - for exploratory analyses of prediction.

HbA1c (long- term blood glucose level), plasma lipid profile, Blood pressure (over day curve); oxygen uptake for assessment of energy expenditure (performed in subgroup only); Body Mass Index; drug use – pharmacological, including antidepressants, smoking and alcohol will be documented.

*Power calculation and statistics*

Power calculation is based on previous studies of motor function after stroke and of the effects of training with HAL. Using FAC as the primary outcome measure and the expected minimum difference of one category between treatment groups, a significance level of 5% and a power of 80%, and an expected minimal loss of patients per treatment arm,

The study will require 54 (18x3) patients at each study sight, i.e. 162 patients in total.

Populations to be used for statistical analyses:

(1) All Available-Patients (AAP) population - all randomised patients regardless of further participation will be used to present demographic and baseline characteristics.

(2) Intent-to-Treat (ITT) population - all patients, who were randomised and started the intervention program regardless if and when they withdrew, will be used to present all efficacy and safety data.

Hypothesis testing will be carried out at the 5% (two-sided) significance level, descriptive summary statistics presented for continuous variables (median, minimum and maximum, arithmetic mean, 95% confidence intervals (CI), standard deviation (SD), standard error).

In non-parametric analyses, the minimum, maximum, median, 5th, 25th, 75th, and 95th percentiles will be calculated. The median difference between treatment groups and the corresponding 95% CI will be presented as statistical analysis outputs. For categorical variables, counts and percentages will be presented, and comparison of intervention groups by use of generalised linear models to provide odds ratios (ORs), corresponding 95% CI and p-values< 0.05. For secondary endpoints we will use mixed models to explore change between and within groups over time.

Adverse events during the study period adverse events (such as falls, skin impact, pain during training session with HAL; new stroke or other cardiovascular events etc.)

will be documented according to a preformed protocol and their association or not to the Interventions will be classified by an external partner. Prevention of these will be standardised over study sites.

*Recruitment period*

Inclusion of patients will be ongoing from September 2015 – October 2017 with follow ups completed in November 2018.

*Limitations, potential bias*

Patients will be recruited from out-patient clinics which enables a recruitment of patients that have been assessed and found to be in need of rehabilitation interventions in the long-term phase after stroke. By using this procedure we will not be able to recruit patients who have not received rehabilitation interventions in out-patient care However, given the access to out-patient rehabilitation units in Stockholm we consider the risk of missing patients who meets the inclusion exclusion critera to be small. Thus, the study samples will represent those with needs of and probably with the greatest potential benefits from improved rehabilitation interventions. Based on our prior experiences, there might be a risk for an uneven gender distribution with more male than female. The reason for this is not clear but women tend to suffer a stroke later in life compared to men. Efforts will be made to avoid a bias in this respect.

*Generalisability*

Regarding the multicentre design, recruitment process and sample size, we expect the findings to be generalisable to the stroke population at focus (see limitiations)

*Funding*

Ongoing exploratory studies are supported by grants. The current project is investigator/academically initiated and driven but with substantial support (See Budget&Resources) from Cyberdyne Inc providing HAL equipment and technical support. The project is otherwise dependent on grants for clinical research as well as contributions from the academic expertise invlolved. Cyberdyne Inc or any other person or body with a financial interest in HAL will not participate in the planning, performance of reporting of the studies. Unlike trials of new pharmacological drugs, there is not tradition or practice for full support from medico technical companies phase III studies of new products, although modern medicine requires similar evidence for technical treatment devices.

Each study site will apply for additional funding as needed (substudies).

*Time plan*

December 2014- September 2015: Coordination of study sites and teams including course for management of the HAL system and standardised performance of assessments and data collection. Submission of study protocols for publication on ClinicalTrials.gov.

September 2015 – October 2017: Inclusion of study participants.

November 2018: Follow up ends.

October 2016 – November 2018: collection of health care utilisation data.

2018-2019 data analyses and reports in relevant international, scientific journals on subsets of data will continue.

*Organisation, study groups, study members/roles, organisations*

This project is based on a consortium for studies of HAL applications in Sweden and Japan (see further below). The groups in Stockholm, Gothenburg and Umeå have collaborated in related areas since long. The group in Umeå represents excellence in research on human motor disorders.

Stockholm: The clinical research group is based on the Department of Rehabilitation Medicine, Danderyd University Hospital (RMD) and members are affiliated to Karolinska Institutet. The group has a main focus on motor disorders after stroke and participates in several projects related to the development, evaluation and implementation of new technology for clinical diagnostics, training and everyday support in neurorehabilitation. The group has an extensive, international network for collaboration and is one partner in projects supported by grants from Vinnova, EU, biotech companies, Promobilia, the Swedish Stroke Association and others.

Jörgen Borg, MD, professor, will serve as chief investigator.

Susanne Palmcrantz, PT, PhD, coordinator of clinical studies related to new technology at RMD will serve as chief coordinator of the studies.

Katarina Skough Vreede, PT, PhD and Anneli Wall, PT, doctoral student, both educated in the HAL technology will serve as tutors and consultants.

Påvel Lindberg PT, PhD, researcher in basic and applied studies of human motor function at Inserm, Paris, and affiliated to Karolinska Institute and RMD. Påvel Lindberg will be responsible for collection and analyses of predictive markers data (e.g. spasticity) in exploratory analyses.

Gothenburg: The study group is involved in studies as part of person centered care and a part of the Stroke Center West. The Stroke Center West endorses the studies as a part of improved stroke care in Western Sweden.

**Katarina Stibrant Sunnerhagen, MD, full professor; has a large network of clinical stroke researches both nationally (through the work on guide lines and as member of the Swedish stroke team council) and internationally (guidelines, VISTA) and the scientific committee for the world stroke conference. She** will serve as principal investigator for the Gothenburg site.

Study coordinator and main applicant: is Susanne Palmcrantz.

Anna Danielsson PT, PhD, has a broad clinical experience and high expertise related to gait and energy expenditure after stroke and will coordinate study activities in Gothenburg.

Umeå: Charlotte Häger, PT, full professor at Umeå University, represents excellence in human motor function and related disability research and will contribute to the supervision of the project, interpretation and reporting of data and will serve as a principle investigator in Umeå.

**Clinical significance**

As outlined above, there is a strong need for new evidence based methods to support recovery of gait function and independence after stroke. The HAL system offers innovative technology designed to match current basic and clinical knowledge about how to optimise training of gait and drive neurobiological processes that may support recovery of functional gait. Further, the kind of training provided by use of the HAL system, may allow not only training of adequate gait pattern but also more intensive physical exercise training, which is one recognised issue not least with regard to secondary stroke prevention.

The HAL system is already used in clinical practice in several neurorehabilitation units in Japan, in accordance with regulations there. Based on previous studies, the HAL system has been CE marked and the method will be available on the market outside Japan. However, introduction of the HAL system in European health care/neurorehabilitation requires evidence from phase III studies of clinical effects and also data on cost effectiveness. With such evidence, we believe that the method will primarily and with no delay, be introduced in specialised departments for rehabilitation medicine/neurorehabilitation and be supported by patient interest groups.

**Health economic considerations**

The economic burden of stroke is huge and the potential significance for the health services is substantial if needs of hospital and/or primary health care and/or community support are reduced.

Improved methods for training of gait early after stroke that improves recovery rate and outcome may have an impact on length of stay in hospital and thus directly reduce costs.

Improved gait and less dependence in everyday life, reduced cardiovascular risk factors/secondary prevention may reduce the need for health care consumption and community support.

**Preliminary results**

Recently, we have reported on the safety and feasibility of the HAL system when used for early onset, intensive gait training as part of an inpatient rehabilitation program for patients with hemiparetic stroke (Nilsson et al 2014, Wall et al 2015). We also reported potential beneficial effects on gait performance in terms of significantly less dependence of others (a change of 1 category on the FAC scale). Further, we also noted that (i) training with HAL seemed to enable and encourage/motivate patients to walk longer distances than would have been expected by conventional training, (ii) study patients rated the experience of training with HAL high and (iii) physiotherapists rated the training procedure as convenient to integrate in the conventional rehabilitation program and potentially time reducing (fewer therapists needed for the same amount of gait training). These observations are in agreement with other studies but must be elaborated on and confirmed in controlled studies.

**National and international collaboration**

This project is based on an established consortium for studies of HAL applications in Sweden and Japan. Members of these groups recently met in Bochum in July 2014 and then agreed on the studies outlined here. The project involves partners in Sweden as presented above and partners in Japan.

The partner in Japan is affiliated to Tsukuba University. The group is lead by professor Sankai, the founder of the HAL system, and includes dr Kawamoto, who is the first or co-author on several publications from the group (see references).

In total, this consortium has an extensive global network within the academia, health care and biotech industry.

**Ethical considerations**

Previous and running studies related to safety, feasibility and effects of training with HAL in Sweden and Germany have been approved by respective Ethical Committees.

There is now consistent evidence from several studies that the HAL method is feasible and safe to use in the specialised stroke rehabilitation context. No major adverse events related to this method have been reported and there is evidence from smaller studies that HAL may be a superior method to improve gait function after stroke. Thus, we find it ethically sound and important to continue according to the plans presented here for phase III studies.

**Equipment**

Basic facilities for the study are provided by the clinical departments.

HAL suits and technical support will be provided by Cyberdyne Inc./Robotdalen Sweden.

Local facilities for substudies (e.g. for gait analyses ) are available at each study site and will be supported by additional applications for funding from other sources (e.g. Promobilia).

Financial support for the new Neuroflexor/foot module is included in this application.

**References**

Bowden MG, Woodbury ML, Duncan PW: Promoting neuroplasticity and recovery

after stroke: future directions for rehabilitation clinical trials. CurrOpinNeurol2013,

26:37–42.

Brazzelli M, Saunders DH, Greig CA, Mead GE. Physical fitness training for stroke patients.

Cochrane Database Syst Rev. 2011 Nov 9;(11).

Dietz V, Muller R, Colombo G. Locomotor activity in spinal man: significance of afferent input from joint and load receptors. Brain. 2002,125:2626-2634.

Dobkin BH, Nadeau SE, Behrman AL, Wu SS, Rose DK, Bowden M, Studenski S, Lu X, Duncan PW Prediction of responders for outcome measures of locomotor Experience Applied Post Stroke trial. J Rehabil Res Dev. 2014;51(1):39-50.

Hesse S, Waldner A, Tomelleri C: Innovative gait robot for the repetitive practice of

floor walking and stair climbing up and down in stroke patients. J Neuroeng Rehabil

2010, 7:30.

Holden MK, Gill KM, Magliozzi MR, Nathan J, Piehl-Baker L. Clinical gait assessment in the neurologically impaired Reliability and meaningfulness. EQ5.PhysTher.1984Jan;64(1):35-40.

Jezernik S, Colombo G, Keller T, Frueh H, Morari M: Robotic Orthosis Lokomat: a

rehabilitation and research tool. Neuromodulation2003, 6:108–115.

Jorgensen HS, Nakayama H, Raaschou HO, Olsen TS. Recovery of walking function in

stroke patients: the Copenhagen Stroke Study. Arch Phys Med Rehabil. 1995,76:27-32.

Kawamoto H, Taal S, Niniss H, Hayashi T, Kamibayashi K, Eguchi K, Sankai Y:

Voluntary motion support control of Robot Suit HAL triggered by bioelectrical signal

for hemiplegia. ConfProc IEEE Eng Med BiolSoc2010:462–466.

Kawamoto H, Kamibayashi K, Nakata Y, Yamawaki K, Ariyasu R, Sankai Y, Sakane M,

Eguchi K, Ochiai N: Pilot study of locomotion improvement using hybrid assistive limb

in chronic stroke patients. BMC Neurol2013, 13:141.

Kwakkel G, Kollen B, Twisk J: Impact of time on improvement of outcome after stroke. Stroke 2006, 37:2348–2353.Langhorne P, Coupar F, Pollock A: Motor recovery after stroke: a systematic review. Lancet Neurol2009, 8:741–754Lindberg PG, Gaverth J, Islam M, Fagergren A, Borg J, Forssberg H. Validation of a New Biomechanical Model to Measure Muscle Tone in Spastic Muscles. NEUROREHABILITATION AND NEURAL REPAIR 2011 25;7 617-625

Liu Z, Zhang RL, Li Y, et al. Remodeling of the corticospinal innervation and spontaneous behavioral recovery after ischemic stroke in adult mice. Stroke 2009; 40:2546–2551.

Mehrholz J, Elsner B, Werner C, Kugler J, Pohl M: Electromechanical-assisted training

for walking after stroke. Cochrane Database Syst Rev 2013, 7.

NILSSON A, VREEDE KS, HAGLUND V, KAWAMOTO H, SANKAI Y, BORG J. Gait training early after stroke with a new exoskeleton - the hybrid assistive limb: a study of safety and feasibility. Journal of neuroengineering and rehabilitation 2014 11; 92

Pekna M, Pekny M, Nilsson M. Modulation of neural plasticity as a basis

for stroke rehabilitation. Stroke 2012; 43:2819–2828.

Pennycott A, Wyss D, Vallery H, Klamroth-Marganska V, Riener R: Towards more effective robotic gait training for stroke rehabilitation: a review. J NeuroengRehabil 2012, 9:65.

Richards LG, Stewart KC, Woodbury ML, et al. Movement-dependent stroke recovery: a systematic review and meta-analysis of TMS and fMRI evidence. Neuropsychologia 2008; 46:3–11.

Stinear C, AckerleyS, Byblow W. Rehabilitation is Initiated Early After Stroke, but Most Motor Rehabilitation Trials AreNot: A Systematic Review. Stroke. 2013;44:2039-2045.

Suzuki K, Mito G, Kawamoto H, Hasegawa Y, Sankai Y: Intention-based walking

support for paraplegia patients with Robot Suit HAL. Adv Robot 2007, 21:1441–1469.

Wall A, Borg J, Palmcrantz S. Clinical application of the Hybrid Assistive Limb (HAL) for gait training - a systematic review. Accepted for publication in Front. Syst. Neurosci. 2015-03-10

Veerbeek JM, van Wegen E, van Peppen R, van der Wees PJ, Hendriks E, Rietberg M, Kwakkel G. [What is the evidence for physical therapy poststroke? A systematic review and meta-analysis.](http://www-ncbi-nlm-nih-gov.proxy.kib.ki.se/pubmed/24505342) PLoS One. 2014 Feb 4;9(2):e87987.

Watanabe H, Tanaka N2, Inuta T, Saitou H, Yanagi H Locomotion Improvement Using a Hybrid Assistive Limb in Recovery Phase Stroke Patients: A Randomized Controlled Pilot Study. Arch Phys Med Rehabil. 2014 Jul 7.

WHO.World Health Report 2003. Geneva: World Health Organization; 2003
